# Supplementary material for: Advancing 3Rs: The Mouse Estrus Detector (MED) as a Low-Stress, Painless, and Efficient Tool for Estrus Determination in Mice
Source: Int J Mol Sci. 2024 Aug 30;25(17):9429. doi: 10.3390/ijms25179429 (PMC11395264; doi:10.3390/ijms25179429)
Supplement: Supplementary file 1 [file ijms-25-09429-s001.zip › ijms-3178437-supplementary.pdf]

Supplementary Materials

Advancing 3Rs: The Mouse Estrus Detector (MED) as a Low-Stress, Painless, and Efficient Tool for Estrus Determination in Mice

Irina V. Belozertseva [1], Dmitrijs D. Merkulovs [2], Helena Kaiser [3], Timofey S. Rozhdestvensky [3,*] and Boris V. Skryabin [3,*]

[1] Valdman Institute of Pharmacology, Pavlov First Saint Petersburg State Medical University, L'va Tolstogo str. 6-8, St. Petersburg 197022, Russia; belozertseva@gmail.com
[2] ELMI Ltd., Bukultu str. 7B, LV-1005 Riga, Latvia; dmitrijs_merkulovs@yahoo.co.uk
[3] Core Facility Transgenic Animal and Genetic Engineering Models (TRAM), Medical Faculty, University of Münster, von-Esmarch str. 56, D-48149 Münster, Germany; helena.kaiser@ukmuenster.de
* Correspondence: rozhdest@uni-muenster.de (T.S.R.); skryabi@uni-muenster.de (B.V.S.)

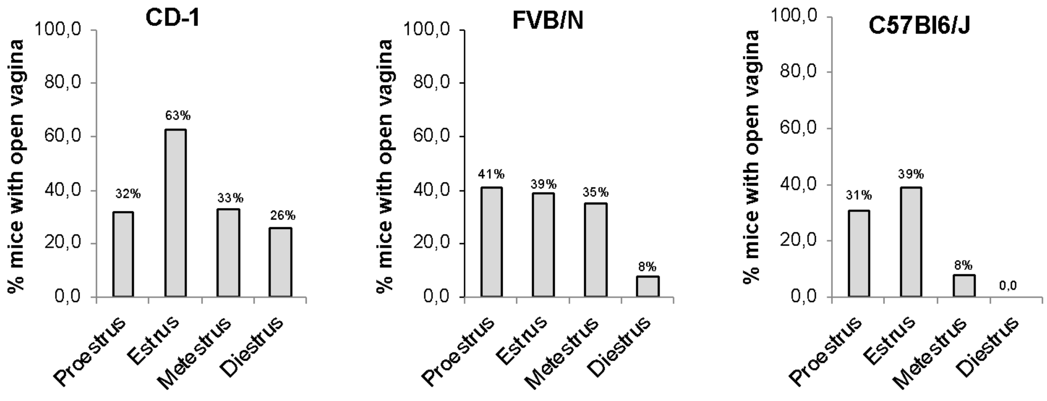

Supplementary Figure S1. Ratio Between Vaginal Opening and Estrous Cycle Determination.

The percentages of animals per strain exhibiting vaginal opening at each stage of the estrous cycle, as determined by vaginal smear cytology, are shown. The total of 110 observations were performed for each strain.

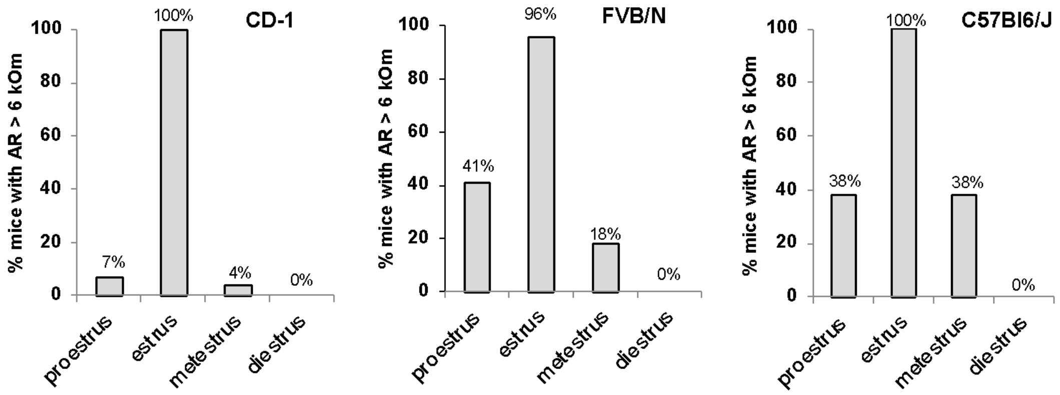

Supplementary Figure S2. Ratio Between Vaginal Active Resistance (AR) measurements and Estrous Cycle Determination.

The percentages of animals per strain exhibiting AR > 6 kOm at each stage of the estrous cycle, as determined by vaginal smear cytology, are shown. The total of 110 measurements were performed for each strain.

Supplementary Table S1. Observational assessments of vaginal opening, AR measurements, and vaginal smear samples cytology (daily evaluations).

Notations:

PE - proestrus; E - estrus; M - Metestrus (Diestrus I); D - diestrus (Diestrus II)

o - open vagina; c - closed vagina.

Strain	Mouse ID	DAY	STAGE (lavage)	AR (kOm) (MEDPro)	Vagina
CD-1	11	1	M	2.8	o
CD-1	11	2	M	2.2	o
CD-1	11	3	P	2.2	c
CD-1	11	4	P	1.6	c
CD-1	11	5	P	1.7	c
CD-1	11	6	P	1.5	c
CD-1	11	7	P	1.5	c
CD-1	11	8	P	1.8	c
CD-1	11	9	E	9.4	o
CD-1	11	10	M	2	c
CD-1	11	11	D	3.7	o
CD-1	12	1	E	24.5	o
CD-1	12	2	M	2.5	o
CD-1	12	3	D	2.7	c
CD-1	12	4	P	9	o
CD-1	12	5	E	10.9	o
CD-1	12	6	E	24	c
CD-1	12	7	M	3	c
CD-1	12	8	D	2.4	c
CD-1	12	9	P	4.7	c
CD-1	12	10	E	8.4	c
CD-1	12	11	M	5.6	o
CD-1	13	1	M	2.4	c

CD-1	13	2	M	2.4	c
CD-1	13	3	P	3.4	c
CD-1	13	4	P	13.9	o
CD-1	13	5	E	8.2	c
CD-1	13	6	M	2.5	c
CD-1	13	7	D	2.8	c
CD-1	13	8	D	2.8	c
CD-1	13	9	D	2.2	c
CD-1	13	10	D	1.4	c
CD-1	13	11	D	1.4	o
CD-1	14	1	E	11.5	c
CD-1	14	2	M	2.2	c
CD-1	14	3	D	3.8	c
CD-1	14	4	M	2.5	o
CD-1	14	5	M	2	c
CD-1	14	6	P	1.8	c
CD-1	14	7	D	1.7	c
CD-1	14	8	D	2.1	c
CD-1	14	9	D	1.4	c
CD-1	14	10	D	0.9	c
CD-1	14	11	P	3	o
CD-1	15	1	D	3.4	c
CD-1	15	2	D	2.1	o
CD-1	15	3	P	2.6	c
CD-1	15	4	E	10	o
CD-1	15	5	M	1.7	c
CD-1	15	6	D	3.2	o
CD-1	15	7	D	3	o
CD-1	15	8	P	4.9	c

CD-1	15	9	P	4.4	c
CD-1	15	10	E	19.5	o
CD-1	15	11	M	2	o
CD-1	21	1	P	2.3	o
CD-1	21	2	E	10.8	o
CD-1	21	3	M	9.8	c
CD-1	21	4	D	2.5	o
CD-1	21	5	D	3.3	c
CD-1	21	6	P	4.5	o
CD-1	21	7	E	16.8	c
CD-1	21	8	M	5.1	c
CD-1	21	9	D	3.1	c
CD-1	21	10	D	2.8	c
CD-1	21	11	P	1.9	o
CD-1	22	1	P	5.6	o
CD-1	22	2	E	20.5	o
CD-1	22	3	M	2.5	o
CD-1	22	4	D	2.5	c
CD-1	22	5	D	2.3	o
CD-1	22	6	P	4.7	c
CD-1	22	7	E	17	o
CD-1	22	8	M	2.2	c
CD-1	22	9	D	2.6	c
CD-1	22	10	D	2.5	c
CD-1	22	11	P	2.4	c
CD-1	23	1	P	2.1	o
CD-1	23	2	E	15.3	o
CD-1	23	3	E	6.5	o
CD-1	23	4	M	3.4	o

CD-1	23	5	D	2.3	c
CD-1	23	6	D	1.5	c
CD-1	23	7	P	5.2	c
CD-1	23	8	E	8.6	o
CD-1	23	9	M	3.5	c
CD-1	23	10	M	2.2	c
CD-1	23	11	D	2.1	c
CD-1	24	1	P	2.5	o
CD-1	24	2	E	19	o
CD-1	24	3	M	1.3	c
CD-1	24	4	D	1.3	c
CD-1	24	5	D	1.1	c
CD-1	24	6	P	0.9	c
CD-1	24	7	E	16	c
CD-1	24	8	M	5.6	c
CD-1	24	9	D	2.3	c
CD-1	24	10	D	2.2	c
CD-1	24	11	D	1.8	o
CD-1	25	1	D	2.2	c
CD-1	25	2	D	1.9	c
CD-1	25	3	P	1.5	c
CD-1	25	4	P	1.1	c
CD-1	25	5	D	1	c
CD-1	25	6	D	2.2	c
CD-1	25	7	P	2.5	c
CD-1	25	8	E	12.9	c
CD-1	25	9	M	1.7	c
CD-1	25	10	D	2.9	o
CD-1	25	11	D	2.6	o

Strain	Mouse ID	DAY	STAGE (lavage)	AR (kOm) (MEDPro)	Vagina
FVB/N	31	1	D	3.8	c
FVB/N	31	2	D	3.2	c
FVB/N	31	3	D	2.5	c
FVB/N	31	4	P	1.7	c
FVB/N	31	5	P	1.8	c
FVB/N	31	6	D	1.8	c
FVB/N	31	7	D	1.7	c
FVB/N	31	8	P	2.6	c
FVB/N	31	9	M	2.7	c
FVB/N	31	10	D	5.4	c
FVB/N	31	11	P	2	c
FVB/N	32	1	D	3	c
FVB/N	32	2	P	3.7	c
FVB/N	32	3	E	9.4	o
FVB/N	32	4	E	6.6	c
FVB/N	32	5	M	6.2	c
FVB/N	32	6	D	4.5	c
FVB/N	32	7	D	2	c
FVB/N	32	8	P	9.2	c
FVB/N	32	9	E	20.3	c
FVB/N	32	10	D	3.7	c
FVB/N	32	11	D	3.2	o
FVB/N	33	1	E	29	o
FVB/N	33	2	M	2.6	o
FVB/N	33	3	D	2.3	c

FVB/N	33	4	D	3.5	c
FVB/N	33	5	P	13.2	o
FVB/N	33	6	E	28.5	c
FVB/N	33	7	M	3.8	o
FVB/N	33	8	D	4.7	c
FVB/N	33	9	P	2.7	c
FVB/N	33	10	E	14.6	c
FVB/N	33	11	E	10	o
FVB/N	34	1	P	8.8	o
FVB/N	34	2	E	30	c
FVB/N	34	3	M	5.2	o
FVB/N	34	4	D	5.2	c
FVB/N	34	5	D	4.1	c
FVB/N	34	6	P	14.5	o
FVB/N	34	7	E	20	c
FVB/N	34	8	E	28	o
FVB/N	34	9	M	2.6	c
FVB/N	34	10	D	3.5	c
FVB/N	34	11	P	4.1	o
FVB/N	35	1	P	4.5	o
FVB/N	35	2	P	21.6	o
FVB/N	35	3	E	30	o
FVB/N	35	4	M	3.5	c
FVB/N	35	5	D	3	c
FVB/N	35	6	D	2.9	c
FVB/N	35	7	P	4.8	o
FVB/N	35	8	E	10.7	c
FVB/N	35	9	E	22.5	c
FVB/N	35	10	M	4.5	c

FVB/N	35	11	D	4.4	c
FVB/N	41	1	E	24.7	c
FVB/N	41	2	M	6.7	o
FVB/N	41	3	D	4.9	c
FVB/N	41	4	D	3.3	c
FVB/N	41	5	D	2.1	c
FVB/N	41	6	P	3.6	c
FVB/N	41	7	E	18.3	o
FVB/N	41	8	M	2.7	c
FVB/N	41	9	D	2	c
FVB/N	41	10	D	2.9	c
FVB/N	41	11	D	2.9	c
FVB/N	42	1	P	16.9	o
FVB/N	42	2	E	28.5	c
FVB/N	42	3	M	5.4	c
FVB/N	42	4	D	3.4	c
FVB/N	42	5	P	9.9	c
FVB/N	42	6	P	12.9	c
FVB/N	42	7	E	23.2	o
FVB/N	42	8	E	7.4	c
FVB/N	42	9	M	6.7	c
FVB/N	42	10	D	3.7	c
FVB/N	42	11	P	5.5	o
FVB/N	43	1	D	3.3	c
FVB/N	43	2	D	2.4	c
FVB/N	43	3	P	3.4	c
FVB/N	43	4	E	22.8	c
FVB/N	43	5	E	9.8	o
FVB/N	43	6	M	2.3	c

FVB/N	43	7	D	2.4	o
FVB/N	43	8	D	2.1	c
FVB/N	43	9	P	6	c
FVB/N	43	10	E	26.2	c
FVB/N	43	11	E	11.2	o
FVB/N	44	1	D	3.6	c
FVB/N	44	2	P	9.5	c
FVB/N	44	3	E	18.2	o
FVB/N	44	4	E	5.3	c
FVB/N	44	5	M	4.5	c
FVB/N	44	6	D	2.8	c
FVB/N	44	7	P	2.5	c
FVB/N	44	8	P	26.8	c
FVB/N	44	9	E	28.4	o
FVB/N	44	10	M	4.8	c
FVB/N	44	11	D	3.3	o
FVB/N	45	1	D	4.7	c
FVB/N	45	2	P	2.6	c
FVB/N	45	3	P	9.6	o
FVB/N	45	4	E	20	c
FVB/N	45	5	M	5.7	o
FVB/N	45	6	D	2.6	c
FVB/N	45	7	P	4.8	o
FVB/N	45	8	E	19.5	c
FVB/N	45	9	E	23.9	c
FVB/N	45	10	M	5.6	o
FVB/N	45	11	D	3.2	c

Strain	Mouse ID	DAY	STAGE (lavage)	AR (kOm) (MEDPro)	Vagina
C57Bl6/J	51	1	M	4.1	o
C57Bl6/J	51	2	M	6	c
C57Bl6/J	51	3	M	2.9	c
C57Bl6/J	51	4	D	2.8	c
C57Bl6/J	51	5	D	2.8	c
C57Bl6/J	51	6	D	2.9	c
C57Bl6/J	51	7	D	5.5	c
C57Bl6/J	51	8	P	14.2	c
C57Bl6/J	51	9	P	5.7	o
C57Bl6/J	51	10	E	20.4	c
C57Bl6/J	51	11	E	22	c
C57Bl6/J	52	1	E	23.2	o
C57Bl6/J	52	2	E	18.4	o
C57Bl6/J	52	3	M	3.8	c
C57Bl6/J	52	4	D	2.6	c
C57Bl6/J	52	5	D	3.5	c
C57Bl6/J	52	6	D	5.9	c
C57Bl6/J	52	7	E	16	c
C57Bl6/J	52	8	E	28.2	c
C57Bl6/J	52	9	E	18.8	o
C57Bl6/J	52	10	M	5.5	c
C57Bl6/J	52	11	D	2.7	c
C57Bl6/J	53	1	P	6.3	c
C57Bl6/J	53	2	E	20.1	c
C57Bl6/J	53	3	E	18.4	c
C57Bl6/J	53	4	M	4.9	c
C57Bl6/J	53	5	D	5.3	c

C57Bl6/J	53	6	P	2.7	c
C57Bl6/J	53	7	E	17.8	o
C57Bl6/J	53	8	E	21.4	c
C57Bl6/J	53	9	E	8.4	o
C57Bl6/J	53	10	E	8.2	c
C57Bl6/J	53	11	M	4.6	c
C57Bl6/J	54	1	P	7.5	c
C57Bl6/J	54	2	E	16.8	c
C57Bl6/J	54	3	M	9.8	c
C57Bl6/J	54	4	D	5.2	c
C57Bl6/J	54	5	D	3.7	c
C57Bl6/J	54	6	P	3	c
C57Bl6/J	54	7	P	13.9	o
C57Bl6/J	54	8	E	19.3	c
C57Bl6/J	54	9	E	17.8	c
C57Bl6/J	54	10	E	19.9	c
C57Bl6/J	54	11	M	5.1	c
C57Bl6/J	55	1	E	17	c
C57Bl6/J	55	2	E	26.3	o
C57Bl6/J	55	3	E	20.3	c
C57Bl6/J	55	4	M	6.1	c
C57Bl6/J	55	5	D	2.8	c
C57Bl6/J	55	6	D	1.8	c
C57Bl6/J	55	7	P	4.3	o
C57Bl6/J	55	8	E	28.1	c
C57Bl6/J	55	9	E	21.9	o
C57Bl6/J	55	10	E	15.1	c
C57Bl6/J	55	11	M	7.2	c
C57Bl6/J	61	1	P	1.6	c

C57Bl6/J	61	2	P	1.5	c
C57Bl6/J	61	3	D	1.6	c
C57Bl6/J	61	4	D	1.8	c
C57Bl6/J	61	5	D	2.1	c
C57Bl6/J	61	6	D	2	c
C57Bl6/J	61	7	D	2.2	c
C57Bl6/J	61	8	D	1.9	c
C57Bl6/J	61	9	D	2.1	c
C57Bl6/J	61	10	D	1.8	c
C57Bl6/J	61	11	D	1.7	c
C57Bl6/J	62	1	M	3.4	c
C57Bl6/J	62	2	M	4.2	o
C57Bl6/J	62	3	D	3.9	c
C57Bl6/J	62	4	D	4.7	c
C57Bl6/J	62	5	D	5.5	c
C57Bl6/J	62	6	D	2.9	c
C57Bl6/J	62	7	E	15	o
C57Bl6/J	62	8	E	12.1	c
C57Bl6/J	62	9	E	7.3	o
C57Bl6/J	62	10	E	11.3	c
C57Bl6/J	62	11	M	1.7	c
C57Bl6/J	63	1	M	6.8	c
C57Bl6/J	63	2	M	5.7	c
C57Bl6/J	63	3	M	5.4	c
C57Bl6/J	63	4	D	3.9	c
C57Bl6/J	63	5	D	4.9	c
C57Bl6/J	63	6	P	3.8	o
C57Bl6/J	63	7	E	9.8	o
C57Bl6/J	63	8	E	30.8	o

C57Bl6/J	63	9	E	25.5	c
C57Bl6/J	63	10	E	13.4	c
C57Bl6/J	63	11	E	28.3	c
C57Bl6/J	64	1	M	5.3	c
C57Bl6/J	64	2	M	8.1	c
C57Bl6/J	64	3	D	3.6	c
C57Bl6/J	64	4	D	4.5	c
C57Bl6/J	64	5	P	15	c
C57Bl6/J	64	6	E	11.8	o
C57Bl6/J	64	7	E	9.8	o
C57Bl6/J	64	8	E	28.4	o
C57Bl6/J	64	9	E	10.7	o
C57Bl6/J	64	10	E	14.5	c
C57Bl6/J	64	11	M	10.4	c
C57Bl6/J	65	1	M	10.1	c
C57Bl6/J	65	2	M	7.6	c
C57Bl6/J	65	3	M	5.2	c
C57Bl6/J	65	4	D	4.2	c
C57Bl6/J	65	5	D	3.3	c
C57Bl6/J	65	6	P	2.7	c
C57Bl6/J	65	7	E	14.9	c
C57Bl6/J	65	8	E	22.5	o
C57Bl6/J	65	9	E	13.6	c
C57Bl6/J	65	10	E	8	c
C57Bl6/J	65	11	M	5.9	c

Supplementary Table S2. The test results for locomotor activity and "emotionality" of Swiss female mice. The tests were conducted on intact female Swiss mice (n=21 - Table S2a) and 30 minutes after either vaginal AR measurements (n=21 - Table S2b) or vaginal lavage collection (n=14 - Table S2c).

Notations:

VER - vertical activity count; HOR - horizontal activity count; AMB - ambulations.

Table S2a

ID	GROUP	Weight	Locomotor activity			fecal boli
			VER	HOR	AMB	
1	intact	36	114	892	442	8
2	intact	36	70	803	357	6
3	intact	29,1	39	807	494	4
4	intact	39,9	36	570	338	11
5	intact	34,2	60	813	531	3
6	intact	38,7	17	436	283	12
7	intact	40,6	153	627	373	7
8	intact	43,6	86	453	257	16
9	intact	34,3	78	533	304	7
10	intact	29,6	72	840	166	13
11	intact	31,1	129	670	428	15
12	intact	31,9	162	584	314	14
13	intact	33,9	95	565	334	18
14	intact	34,7	83	638	338	12
15	intact	38,1	10	387	221	6
16	intact	35,7	50	672	318	11
17	intact	40,4	162	757	434	9
18	intact	31,8	75	427	209	6
19	intact	34,3	41	534	313	14
20	intact	29,8	54	520	305	6
21	intact	39,2	64	696	410	9

Table S2b

ID	GROUP	Weight	Locomotor activity			fecal boli
			VER	HOR	AMB	
101	MEDPro	41,1	136	348	199	11
102	MEDPro	47,2	132	528	241	7
103	MEDPro	34,3	58	496	266	11
104	MEDPro	33	58	1175	813	9
105	MEDPro	32,7	71	653	356	7
106	MEDPro	27	121	1010	647	5
107	MEDPro	34,6	79	444	237	13
108	MEDPro	32,4	55	446	260	6
109	MEDPro	35,2	210	466	251	7
110	MEDPro	36,3	222	432	253	9
111	MEDPro	40,7	28	352	225	4
112	MEDPro	33,7	147	710	489	12
113	MEDPro	26.9	16	390	229	6
114	MEDPro	30.9	81	379	205	9
115	MEDPro	37,9	89	435	238	6
116	MEDPro	38,2	88	660	273	4
117	MEDPro	32	75	526	293	15
118	MEDPro	37,3	79	526	237	10
119	MEDPro	36,3	100	476	259	4
120	MEDPro	35,3	188	479	269	7
121	MEDPro	35,5	198	438	265	6

Table S2c

ID	GROUP	Weight	Locomotor activity			fecal boli
			VER	HOR	AMB	
201	lavage	45,6	85	310	157	11
202	lavage	47,1	155	754	432	13
203	lavage	44	6	237	98	13
204	lavage	46,1	122	450	259	10
205	lavage	32,6	72	464	248	13
206	lavage	33,2	69	650	398	10
207	lavage	34	30	376	203	8
208	lavage	36,6	46	365	173	11
209	lavage	29,8	103	722	395	12
210	lavage	33,8	17	470	267	16
211	lavage	30,4	19	445	236	3
212	lavage	33,3	15	446	243	12
213	lavage	37,8	89	611	364	7
214	lavage	37,3	2	176	78	11
